# iMSAT: a novel approach to the development of microsatellite loci using barcoded Illumina libraries

**DOI:** 10.1186/1471-2164-15-858

**Published:** 2014-10-04

**Authors:** Jeremy C Andersen, Nicholas J Mills

**Affiliations:** Department of Environmental Science Policy and Management, University of California Berkeley, Wellman Hall, Berkeley, USA

## Abstract

**Background:**

Illumina sequencing with its high number of reads and low per base pair cost is an attractive technology for development of molecular resources for non-model organisms. While many software packages have been developed to identify short tandem repeats (STRs) from next-generation sequencing data, these methods do not inform the investigator as to whether or not candidate loci are polymorphic in their target populations.

**Results:**

We provide a python program iMSAT that uses the polymorphism data obtained from mapping individual Illumina sequence reads onto a reference genome to identify polymorphic STRs. Using this approach, we identified 9,119 candidate polymorphic STRs for use with the parasitoid wasp *Trioxys pallidus* and 2,378 candidate polymorphic STRs for use with the aphid *Chromaphis juglandicola*. For both organisms we selected 20 candidate tri-nucleotide STRs for validation. Using fluorescent-labeled oligonucleotide primers, we genotyped 91 female *T. pallidus* collected in nine localities and 46 female *C. juglandicola* collected in 4 localities and found 15 of the examined markers to be polymorphic for *T. pallidus* and 12 of the examined markers to be polymorphic for *C. juglandicola*.

**Conclusions:**

We present a novel approach that uses standard Illumina barcoding primers and a single Illumina HiSeq run to target polymorphic STR fragments to develop and test STR markers. We validate this approach using the parasitoid wasp *T. pallidus* and its aphid host *C. juglandicola*. This approach, which would also be compatible with 454 Sequencing, allowed us to quickly identify markers with known variability. Accordingly, our method constitutes a significant improvement over existing STR identification software packages.

**Electronic supplementary material:**

The online version of this article (doi:10.1186/1471-2164-15-858) contains supplementary material, which is available to authorized users.

## Background

Next-Generation Sequencing (NGS) technologies have recently revolutionized the ease and the rate at which genetic resources can be developed [1–3]. This revolution has made it possible to now use the genetic tools in nearly all organisms that were previously only available for model taxa [4]. For example, the development of short tandem repeat (STR or microsatellite) markers in non-model organisms is currently undergoing a complete paradigm shift in regards to the techniques and methods used to isolate potential markers, particularly for insects [5–8]. These new techniques have replaced the laborious steps of DNA cloning with the speed and ease of NGS technologies [9] allowing researchers to quickly develop candidate markers for their study organisms.

Perhaps as a result of the increased accessibility to NGS technologies for STR marker development, there has also been an increased level of activity in the development of associated software for identifying candidate markers. Many highly cited packages exist [10–22], but see [23] for a more thorough review. Current software packages work by searching through assembled sequence data for tandem-repeat regions, and then apply filters to optimize the list of candidate sites based on user specified criteria. Newer programs directly allow for the use of whole genome data [21] or raw sequence data from paired-end Illumina sequencing [22]. The most recent software program, SSR_pipeline, represents a particularly important improvement in the identification of STR data by directly using quality scores from the sequence reads to aid in the identification of STR markers. Yet, two major obstacles remain for the identification of STR markers for genetic analyses based on NGS sequence results. First, most existing software packages provide an overwhelming number of candidate loci. Second, they do not inform the investigator as to which loci are polymorphic for the populations under study. For example, a recent study that integrated NGS technologies with existing software packages to develop markers for a species of aphid [24] found that only 0.76% of their 342 candidate markers were suitable for use, though whether this was due to failure to amplify target loci with standard PCR protocols or because amplified loci were not polymorphic is unknown to us.

To improve the rate at which polymorphic STR markers can be identified and developed for use in genetic analyses, we present the use of a novel technique that uses barcoded Illumina sequencing libraries to identify polymorphic STR markers. We test this technique using two insect species from phylogentically distinct orders: the braconid wasp *Trioxys pallidus* and its aphid host *Chromaphis juglandicola.* Both insects occur in walnut orchards in California where *C. juglandicola* is an important invasive pest that was brought under effective biological control by the deliberate introduction of *T. pallidus* from Iran in 1969 [25–27]. We then compare the patterns of STR motifs found for each species to other results published from their respective orders to examine the value of this approach for phylogenetically diverse organisms.

## Results

### Next-generation sequencing results

Our Illumina sequencing run for *T. pallidus* resulted in over 99 million 100 base pair reads and our Illumina sequencing run for *C. juglandicola* resulted in over 170 million 100 base pair reads. Using the *de novo* genome assembly program Velvet [28], we constructed 65,535 contigs with an average length of 834.2 base pairs and an average coverage of 8.0X for *T. pallidus*. For *C. juglandicola*, we developed 474,388 contigs with an average length of 2,573 base pairs and an average coverage of 11.2X. Raw sequence reads were uploaded to BioSample (Accession Numbers SAMN03020618 - SAMN03020621).

### Comparison of iMSAT to other methods for identifying STRs

Using MSATCOMMANDER [12] and Phobos [20], we identified 18,525 and 21,860 STRs for *T. pallidus* (Table 1) and 187,270 and 100,290 STRs for *C. juglandicola* (Table 2), respectively. Using our novel python program iMSAT (https://sourceforge.net/projects/imsat/), we found 9,119 candidate polymorphic STRs for *T. pallidus* (Table 1) and 2,378 candidate polymorphic STRs for *C. juglandicola* (Table 2). For *T. pallidus* di-nucleotide STRs were the most abundant type identified by all three methods in being between 55% and 65%, tri-nucleotide STRs represented between 31% and 38% and tetra- and penta-nucleotide STRs constituted between 4% and 6% combined. For *C. juglandicola,* di-nucleotide STRs were again the most abundant type identified by all three methods (82-93%). However, for this species, tri-nucleotide STRs were rare (4.6-17%) while tetra- and penta-nucleotide STRs were extremely rare (0-3%).

**Table 1:** **STR results from** ***Trioxys pallidus***

	A) Phobos				B) MSATCOMMANDER				C) iMSAT			
repeats	di	tri	tetra	penta	di	tri	tetra	penta	di	tri	tetra	penta
5	4132	1612	262	38	5338	3496	657	44	772	500	93	18
6	3737	3317	683	38	2090	1898	218	4	1718	837	145	8
7	1751	1788	229	6	1243	980	59	2	1181	717	80	1
8	1104	958	64	2	762	417	28	1	765	426	29	1
9	616	379	29	1	411	151	10	0	410	178	9	0
10	355	133	12	0	240	46	9	0	228	72	1	0
11	194	43	8	0	134	19	2	0	177	41	2	0
12	105	20	3	0	60	22	0	0	129	17	1	0
13	52	18	0	0	29	4	0	0	92	5	0	0
14	19	5	1	0	13	10	1	0	64	9	0	0
15	15	9	0	0	15	5	6	0	65	5	0	0
16	7	7	8	0	3	3	2	0	46	1	0	0
17	3	4	0	0	1	4	0	0	51	5	0	0
18	1	3	1	0	5	7	0	0	20	0	0	0
19	5	6	0	0	3	16	0	0	39	0	0	0
20	3	17	0	0	1	34	0	0	36	0	0	0
21+	16	41	0	0	16	7	0	0	124	1	0	0
SUM	12115	8360	1300	85	10363	7119	992	51	5917	2814	360	28
Percent	55.4	38.2	5.9	0.3	55.9	38.4	5.4	0.3	64.9	30.9	3.9	0.3

Results comparing the total numbers of discovered repeats for each pattern type (di, tri, tetra, or penta) using Phobos, MSATCOMMANDER, and iMSAT. The total numbers of repeats for each pattern are summed, and presented as a percentage of total repeats found using each software program.

**Table 2:** **STR results from** ***Chromaphis juglandicola***

	A) Phobos				B) MSATCOMMANDER				C) iMSAT			
repeats	di	tri	tetra	penta	di	tri	tetra	penta	di	tri	tetra	penta
5	21729	7100	347	29	35052	16556	890	33	9	8	3	0
6	12739	4282	123	1	22177	9949	326	4	39	7	14	0
7	8973	2628	37	1	16641	5961	133	3	63	12	13	0
8	7009	1546	12	1	13107	3231	65	2	149	30	11	0
9	5416	848	6	0	9805	1580	38	3	275	25	4	0
10	3860	389	6	1	6850	754	26	0	344	12	10	0
11	2746	193	2	0	4844	382	11	0	260	6	5	0
12	1890	97	2	0	3282	173	4	0	244	2	2	0
13	1283	39	2	0	2403	95	9	0	164	2	4	0
14	937	14	2	0	1875	50	9	0	133	5	0	0
15	793	12	1	0	1518	32	7	0	100	1	0	0
16	709	3	1	0	1433	14	5	0	81	0	0	0
17	608	4	0	0	1205	9	1	0	84	0	0	0
18	577	3	0	0	1283	8	0	0	53	0	0	0
19	626	2	0	0	1228	10	0	0	44	0	0	0
20	604	5	0	0	1211	2	0	0	35	0	0	0
21+	12028	24	0	0	16100	18	0	0	125	0	0	0
SUM	82527	17189	541	33	146877	38824	1524	45	2202	110	66	0
Percent	82.3	17.1	0.5	0.03	78.4	20.7	0.8	0.02	92.6	4.6	2.8	0

Results comparing the total numbers of discovered repeats for each pattern type (di, tri, tetra, or penta) using Phobos, MSATCOMMANDER, and iMSAT. The total numbers of repeats for each pattern are summed, and presented as a percentage of total repeats found using each software program.

### Amplification of tri-nucleotide STRs in *T. pallidus*

Of the selected 20 STRs from our output of candidate polymorphic tri-nucleotide STRs, we consistently amplified 17 of them with standard PCR protocols. Two of these markers, TpMSAT3 and TpMSAT6 were excluded from the analysis because they displayed repeat patterns not consistent with tri-nucleotide STRs. DNA sequences for the repeat region of each STR marker used in this study were uploaded to GenBank (Accession #’s KC477413 - KC477427) and their characteristics were summarized in Table 3.

**Table 3:** **Characteristics of the 15 and 12 polymorphic STRs isolated from** ***T. pallidus*** **and** ***C. juglandicola***

Locus	Repeat motif	Fragment lengths	***T***_***A***_	***N***_***A***_	P _HWE_	GenBank accession
*T. pallidus*						
TpMSAT01	(ATC)_14–18_	366 – 378	57	5	0.260	KC477413
TpMSAT02	(ATC)_6–20_	345 – 387	57	7	0.918	KC477414
TpMSAT04	(CGA)_4–10_	475 – 493	57	7	0.742	KC477415
TpMSAT05	(TGA)_15–18_	330 – 336	57	3	0.037	KC477416
TpMSAT07	(CAG)_4–19_	322 – 370	57	6	0.324	KC477417
TpMSAT08	(GAC)_5–10_	305 – 320	57	5	0.808	KC477418
TpMSAT09	(TAC)_3–9_	294 – 312	57	5	0.066	KC477419
TpMSAT10	(GCT)_2–8_	396 – 414	57	7	0.093	KC477420
TpMSAT11	(TCA)_4–10_	300 – 336	50	7	0.480	KC477421
TpMSAT12	(AAC)_5–9_	255 – 267	57	5	0.478	KC477422
TpMSAT13	(TCA)_3–16_	422 – 461	57	8	0.002*	KC477423
TpMSAT14	(AAG)_3–11_	313 – 340	57	9	0.147	KC477424
TpMSAT16	(TGA)_12–16_	317 – 329	57	5	0.273	KC477425
TpMSAT17	(ATT)_6–15_	340 – 367	57	6	0.940	KC477426
TpMSAT19	(GAA)_4–13_	260 – 287	57	6	0.164	KC477427
*C. juglandicola*						
CjMSAT01	(TAA)_10–11_	210 – 213	50	2	NA	KJ939575
CjMSAT02	(CAA)_9–16_	375 – 396	50	5	0.090	KJ939576
CjMSAT03	(TAC)_11–12_	374 – 377	50	2	NA	KJ939577
CjMSAT04	(TAC)_18–21_	347 – 356	50	4	1	KJ939578
CjMSAT05	(ATA)_10–12_	291 – 297	50	3	1	KJ939579
CjMSAT08	(TAA)_0–15_	239 – 284	57	2	NA	KJ939580
CjMSAT09	(TAA)_9–10_	276 – 279	50	2	NA	KJ939581
CjMSAT13	(CGT)_10–18_	264 – 288	50	7	NA	KJ939583
CjMSAT14	(ATT)_10–13_	460 – 469	50	3	0.247	KJ939584
CjMSAT16	(ATA)_7–8_	367 – 370	57	2	NA	KJ939585
CjMSAT18	(ATT)_10–12_	320 – 326	50	3	1	KJ939586
CjMSAT19	(TAC)_14–15_	318 – 321	50	2	NA	KJ939587

STR name, repeat motif, fragment lengths of observed alleles, annealing temperature in degrees Celsius (*T*_*A*_), number of observed alleles (*N*_*A*_), P values from Hardy-Weinberg Equilibrium statistics (P_HWE_), and GenBank accession numbers.

*Indicates a significant deviation from HWE after applying Bonferroni’s correction for multiple-comparison.

### Characteristics of STR markers in *T. pallidus*

Allelic diversity ranged from three alleles per locus for TpMSAT05 to nine for TpMSAT11 and TpMSAT14 (Table 3). Measures of averaged heterozygosity ranged from 0.21 to 0.54 for Ho and 0.33 to 0.54 for He (Table 4). One locus, TpMSAT05, exhibited a marginally significant deviation from Hardy-Weinberg Equilibrium (HWE) (*χ*^2^ = 16.44, DF = 8, P = 0.04), though this deviation was not significant after Bonferroni correction for multiple comparisons (corrected α = 0.013). Another locus, TpMSAT13, exhibited a highly significant deviation from HWE (*χ*^2^ = 40.23, DF = 8, P = 0.002), which was still significant after Bonferroni correction (corrected α = 0.006). Linkage disequilibrium was not observed between any of the STR markers.

**Table 4:** **Source populations of** ***T. pallidus*** **and** ***C. juglandicola***

Pop	Location	Host	Collector	Date	N	Ho	He
*T. pallidus*							
J0029	Bethel, OR	*M. coryli*	J Andersen and C Hedstrom	24vi2010	6	0.544	0.537
J0030	McMinnville, OR	*M. coryli*	J Andersen	24vi2010	6	0.208	0.412
J0001	Durham, CA	*C. juglandicola*	N Mills	06vii2006	12	0.311	0.328
J0008	Tulare, CA	*C. juglandicola*	N Mills	17ix2006	15	0.271	0.373
J0069	Upper Lake, CA	*C. juglandicola*	R Elkins	10ix2010	11	0.312	0.385
J0178	Yuba City, CA	*P. juglandis*	J Andersen	27ix2011	7	0.242	0.360
J0179	Escalon, CA	*C. juglandicola*	J Andersen and M Labbé	05vi2012	12	0.344	0.354
J0188	Newark, CA	*C. juglandicola*	J Andersen and M Labbé	30viii2012	10	0.347	0.384
J0163	Tehran, Iran	*C. juglandicola*	P Starý	24iii2004	12	0.321	0.381
*C. juglandicola*							
A0046	Modesto, CA	Walnut	J Andersen and K Anderson	7vii2010	9	0.103	0.100
A0052	Linden, CA	Walnut	J Andersen	10vii2010	8	0.112	0.128
A0073	Upper Lake, CA	Walnut	J Andersren and M Labbé	13ix2010	9	0.151	0.165
A0164	Parnac, France	Walnut	J Andersen and M Labbé	2vi2011	20	0.068	0.082

Populations used in this study including the number of females genotyped (N), averaged observed (Ho), and expected (He) heterozygosity.

### Amplification of tri-nucleotide STRs in *C. juglandicola*

We selected 20 STRs from our output of candidate polymorphic tri-nucleotide STRs and we were able to consistently amplify 16 of them with standard PCR protocols. Of the 16 markers all but three were found to be polymorphic in our sample populations. One of these markers, CjMSAT12, was excluded from the analysis because it displayed fragment length polymorphisms outside of its expected range. DNA sequences for the repeat region of each STR marker used in this study were uploaded to GenBank (Accession #’s KJ939575 - KJ939587), and their characteristics were summarized in Table 4.

### Characteristics of STR markers in *C. juglandicola*

Allelic diversity for polymorphic loci ranged from two alleles per locus for CjMSAT01, CjMSAT03, CjMSAT08, CjMSAT09, CjMSAT16, and CjMSAT19 to seven for CjMSAT13. Measures of averaged heterozygosity ranged from 0.08 to 0.15 for Ho and 0.08 to 0.17 for He (Table 4). No locus displayed deviations from HWE, and there was no evidence of linkage disequilibrium observed between any of the STR markers.

## Discussion

The genomic revolution sparked by the advent of NGS is well underway, and its low per base pair cost and high number of sequence reads yields many benefits and tools [29], including the rapid development of polymorphic markers for population genetic studies. Our pipeline involving iMSAT identifies polymorphic STRs from two simultaneously obtained sequencing reads. The output of iMSAT facilitates the design of primers for population-level studies, reducing the time and expense associated with the production of STRs.

### Potential benefits and limitations

iMSAT represents a significant improvement over existing techniques. NGS technologies are today’s standard for developing STR markers e.g. [5, 30]. They have eliminated the laborious steps associated with plasmid cloning [reviewed by [31]]. However, candidate markers still require testing to identify polymorphic regions. Given the large numbers of candidate markers identified by existing software packages (Tables 1 and 2), the selection of candidate polymorphic loci and their validation with PCR is both expensive and time consuming. Two recent studies using NGS technologies and existing STR software programs employed PCR screening to examine 48 [32] and 342 [24] candidate markers, for which only 11 (23%) and 26 (0.76%), respectively, were used in the subsequent studies. Whether these relatively low rates of success in the development of effective markers were due to problems with PCR amplification or to fixation of the markers once amplified is unclear. Regardless, our approach identifies 17 of the 20 candidate polymorphic STRs, among which 75% of the original 20 are polymorphic for *T. pallidus.* Thus, iMSAT is a useful tool for population genetic research. Similarly, 16 out of 20 candidates amplified consistently and 12 of the 16 markers (60% of the original 20) are polymorphic for *C. juglandicola*.

Our program adds virtually no costs to the overall production of STR markers, as an additional Illumina Sequencing Library, for example, can be produced by a third party for as little as $200 USD (quote from the Functional Genomics Laboratory at the University of California Berkeley, June 2014). Freely available, iMSAT generates a list of polymorphic STR markers in a fast and cost-effective manner. Although iMSAT recovers far fewer potential STR markers than other programs, its ability to identify candidate polymorphic greatly outweighs the reduction in total numbers of potential markers. Most studies based on STRs have used relatively few markers (10–50) and, statistically, there is no need to develop upwards of 12,000 STR markers that is possible using NGS technologies. The ‘novelty’ of our approach is to use the polymorphism data provided by the raw sequence reads themselves to identify candidate STR markers, and our program takes advantage of the output from existing software tools [28, 33, 34].

A similar approach to screening NGS sequence results for polymorphic regions before STR development has previously been presented by Hoffman and Nichols [35]. These authors also pooled DNA extracts to create a single sequencing library for 454 sequencing, re-mapped the individual sequence reads to their *de novo* assembly, and targeted STR repeats that appeared polymorphic. While similar in that both approaches perform *in silico* polymorphism detection, ours has the advantages that by using barcoded libraries we were able to assign sequence reads to both of our populations of *T. pallidus* and *C. juglandicola* with only a single run each. This advantage is particularly valuable, as it allows the identification of markers that not only are likely to be polymorphic, but whose polymorphism can also be characterized as either within and/or between populations. This greatly increases the utility of the data in generating useful STR markers, and may in part explain the greater rate of success we observed in isolating polymorphic markers.

### Comparison of results with other species of insects

The availability of published genomes from several insect species allows for comparative genomic analyses, including examinations into the diversity and distribution of STR motifs. Behura and Severson [36] compared coding sequences from 25 species of insects representing five different orders. In contrast to our findings for *T. pallidus* and *C. juglandicola*, they found that tri-nucleotide repeats were the most common repeat type across insects. Their results may be inherently biased towards recovery of tri-nucleotide repeats, however, because they focused on coding regions of DNA where single or double base pair insertions/deletions are unlikely [37]. Another recent study [38] examined both coding and non-coding regions, compared published whole genome sequence data from 12 species of insects representing six orders. Although most species had predominantly di- or tri-nucleotide repeats, no one type was dominant; even congeners differed in which type of repeats were most abundant. The most dramatic example occured between *Drosophila simulans* and *D. melanogaster*. While *D. simulans* had relatively equal proportions of di-, and tri-nucleotides as the most abundant repeat types, penta-nucleotide repeats were most abundant in *D. melanogaster* and twice more than any other type of repeat. Interestingly, they found that STRs were more common among the Hymenoptera and represented a higher percentage of the genome than in any of the other orders of insects examined. The Hymenoptera also differed from other orders in that di-nucleotide repeats were the most abundant type of repeat – between 2 and 5 times more frequent than tri-nucleotide repeats. For the aphid *Acyrthosiphon pisum,* di-nucleotide repeats were about half as abundant as in their examined hymenopteras. Contrary to our results for *C. juglandicola,* they also found that tri-nucleotide repeats were the most abundant type of repeat unit.

## Conclusions

We announce a novel approach for using NGS technologies in conjunction with several popular software packages to identify polymorphic STRs. This approach allows the rapid and cost-effective development of 15 polymorphic STRs for *T. pallidus* and 12 for *C. juglandicola.*

## Methods

To identify and test STR markers, we used an NGS approach. Sequencing libraries for *T. pallidus* were created by pooling twenty individuals of *T. pallidus* reared from filbert aphids collected in Bethel, Oregon, United States into a sample labeled “Hazelnut”, and twenty individuals of *T. pallidus* reared from walnut aphids collected in Tehran, Iran into a sample labeled “Walnut”. DNA was then extracted from each pooled sample using a Qiagen DNeasy Blood & Tissue Kit (Qiagen) with the following modification. To reduce the amount of residual salt in the extract, critical for NGS applications, we performed the AW1 and AW2 washes twice each, followed by an additional spin step to remove any residual AW2 buffer. This was followed by standard elution with the AE buffer. Sequencing libraries for *C. juglandicola* were created by pooling 20 individuals of *C. juglandicola* collected in Upper Lake, California, United States, into a sample labeled “US”, and 20 individuals of *C. juglandicola* collected in Parnac, France into a sample labeled “France”. DNA was then extracted from each pooled sample using the Qiagen Gentra-PureGene DNA Extraction Kit (Qiagen). Concentrations of nucleic acids for all extracts were then quantified with a ND-1000 NanoDrop® (NanoDrop Technologies, Inc.) and concentrations of double stranded DNA were measured using the Qubit® dsDNA HS Assay kit (Life Technologies Corp.). Sequencing libraries for each *T. pallidus* extract were created using the Nextera™ DNA Sample Prep Kit (Illumina, Inc.) as per instructions, and each library was constructed using a different Illumina barcoding primer. Sequencing libraries for each *C. juglandicola* extract were created using the PrepX™ ILM DNA Library Kit (Wafergen Biosystems, Inc.) at The Functional Genomics Laboratory of the University of California Berkeley and each library was constructed using a different Illumina barcoding primer. Sequencing libraries were examined for fragment length distribution and concentrations using a 2100 Expert Bioanalyzer (Agiliant Technologies), and a KAPA Biosystems Library Quantification Kit (KAPA Biosystems). Each species’ libraries were then pooled together, and sequenced independently each using a single run of an Illumina HiSeq2000 (Illumina, Inc.) sequencer at the Vincent J. Coates Genomics Sequencing Laboratory, University of California Berkeley.

Summary statistics representing the sequence results from the Illumina HiSeq2000 run were calculated using the FASTX-Toolkit [39] and this program was then used to filter low quality reads. Individual Illumina sequencing reads were then assembled into contigs using the *de novo* assembly program Velvet 1.1.06 [28] with a kmer length of 65 for *T. pallidus* and 67 for *C. juglandicola*. We then used MSATCOMMANDER [12] and Phobos [20] to identify di-, tri-, tetra-, and penta-nucleotide repeat patterns with their default settings. We then compared these results to those identified with iMSAT, our novel python program (https://sourceforge.net/projects/imsat/). iMSAT uses a “.vcf” report file of polymorphic sites generated from mapping NGS sequencing reads to a genome assembly using BWA [34] and SAMtools [33]. Both BWA and SAMtools are widely used for the identification and analysis of single nucleotide polymorphisms [40–42].

**Figure 1:**
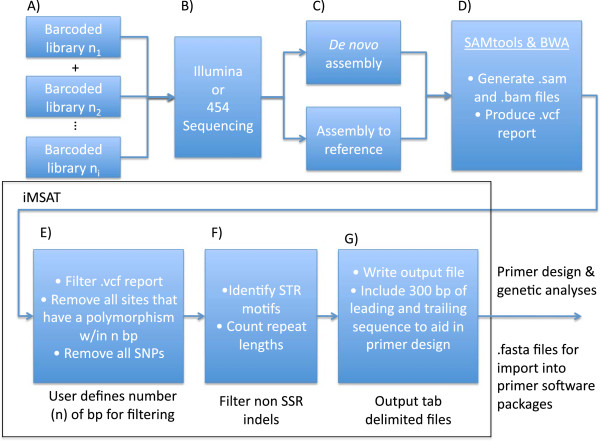
**iMSAT workflow diagram.** Before using iMSAT, barcoded NGS sequencing libraries are produced **(A)** and sequenced **(B)** and either used to create a *de novo* assembly or to align an available reference genome **(C)**. Then using SAMtools and BWA the individual sequence reads are used to create a polymorphism report **(D)** that includs the location of the polymorphic loci, type (SNP or INDEL), and other quality statistics. iMSAT then uses the output and the alignment file to filter the polymorphism data based on a user specified number of base pairs **(E)**, identifies the STR motifs and the number of repeats **(F)**, and outputs separate .FASTA preprocessing files for each candidate locus that can be used with primer design software **(G)**.

iMSAT uses an interactive command-line interface (see Figure 1 for a graphical representation). The first user prompt asks for the locations of the alignment and “.vcf” files as well as the formatting of the alignment file. iMSAT can process alignment files with both traditional “.FASTA preprocessing” formatted sequence data (i.e. one line beginning with a “>” followed by the sequence name, and a second line with the sequence data) or a tab-delimited format (i.e. one line with both sequence name and sequence data separated by a tab). Our program subsequently filters the “indel” data from the “.vcf” report, and searches for all polymorphic sites that represented di-, tri-, tetra-, and penta-nucleotide STRs that were greater than five repeat units in length. The user is prompted as to whether or not they would like a separate list of polymorphic STR markers that are “fixed” in one of their target populations. The program then produces a “.FASTA preprocessing” formatted file identifying the location of the polymorphic STR in the sequence title and 300 bp of both the leading and trailing sequence strands to allow for the production of primers. Sequence information for all primers used, including fluorescent label are available in Additional file 1.

For *T. pallidus,* we tested the program as if whole genome assembly was being used. To do so, we combined the contig sequences generated by Velvet [28] into one continuous DNA sequence strand with the union of two contig sequences being differentiated by the addition of 100 “N” base pairs. The addition of these “N” base pairs ensured that when we could exclude any potential STR markers that would be artificially created when we joined the separate consensus sequences. For *C. juglandicola* we tested the program using the raw output from Velvet [28] where all 474,388 contigs were represented in traditional FASTA preprocessing formatting. For both species we then used the “vcf” report generated using BWA [34] and SAMtools [33] to target polymorphic STRs.

To validate this approach, we filtered the data to only include those repeat regions that were; a) tri-nucleotide repeats, b) were composed of high-quality reads based on the “vcf” file, and c) had no “N” base calls within 300 base pairs of the repeat region to allow for primer construction. Though the majority of candidate STRs were di-nucleotide repeats, we selected tri-nucleotide repeats because of the known problems associated with scoring di-nucleotide STRs caused by “stutters” [43]. For each species, we then selected the 20 tri-nucleotide candidate markers with the greatest number of repeat units. Primer pairs for all markers were generated using Primer3 [44] as implemented in Geneious 5.6.2 [45]. To ease multiplexing, primers were designed to be at least 20 base pairs in length and to have an optimal annealing temperature of 57°C.

To test the candidate markers, DNA was extracted from 91 female *T. pallidus* reared from three species of aphid (*C. juglandicola* and *Panaphis juglandis* on walnut, and *M. coryli* on filbert) from nine different localities (Table 4), and from 46 female *C. juglandicola* from four different localities (Table 4) using the Qiagen DNeasy Blood & Tissue Kit (Qiagen). Non-labeled oligonucelotide primers were used to test and optimize the conditions of each of the 20 candidate regions for each species through standard PCR protocols and the amplified fragments were sequenced at the DNA Sequencing Facility of the University of California Berkeley. For candidate markers that were consistently amplified, fluorescent-labeled primers compatible with the GeneScan™ 600 LIZ size standard (Life Technologies) were used. PCR conditions were then re-optimized for the fluorescent-labeled primers. For both species markers were amplified using one of two PCR protocols signified by their primary annealing temperature (T_a_ 57 or T_a_ 50). For T_a_ 57 an initial denaturation for 5 min at 95°C was followed by 35 cycles of 95°C for 1 min, 57°C for 1.5 min, 72°C for 1 min, followed by a 10 min extension period at 72°C. For T_a_ 50, a touchdown protocol was used with the following profile: an initial denaturation for 5 min at 95°C, followed by 14 cycles of 95°C for 1 min, 57°C for 1.5 min, and 72°C for 1 min where the annealing temperature decreased 0.5°C every cycle, followed by 30 cycles with an annealing temperature of 50°C, and a 10 min extension period at 72°C.

Fragment lengths were measured in comparison to the GeneScan™ LIZ® 600 Size Standard v. 2.0 (Life technologies) using an Applied Biosystems 3730XL (Life Technologies) at the DNA Sequencing Facility at the University of California Berkeley, and scored using the Microsatellite Plug-in for Geneious 5.6.2 [45]. The number of alleles per locus (*k*), averaged observed (Ho) and expected (He) heterozygosity, deviations from Hardy-Weinberg equilibrium (HWE), and presence of linkage disequilibrium (LD) between loci were examined using GenePop 4.2 [46, 47].

## Availability of supporting data

The data sets supporting the results of this article are available in the NCBI data repository. Raw sequence reads from the Illumina HiSeq runs for *Trioxys pallidus* and *Chromphis juglandicola* have the following Accession Numbers: SAMN03020618 - SAMN03020621 and can be found at http://www.ncbi.nlm.nih.gov/biosample/.

Sequences of SSR loci for *Trioxys pallidus* have the following Accession Numbers: KC477413 - KC477427 and can be found at http://www.ncbi.nlm.nih.gov/nuccore/.

Sequences of SSR loci for *Chrompahis juglandicola* have the following Accession Numbers: KJ939575 - KJ939587 and can be found at http://www.ncbi.nlm.nih.gov/nucore/.

The python script and supporting information are available in the SourceForge source code repository at http://sourceforge.net/projects/imsat/.

## Electronic supplementary material

Additional file 1: **STR primer sequences.** Excel spreadsheet including the sequence data for forward and reverse primers used to target each STR marker. (XLS 30 KB)
